# Prevalence of *Legionella* in a Public Building Water Plumbing System During COVID-19 Lockdown

**DOI:** 10.1021/envhealth.3c00058

**Published:** 2023-10-04

**Authors:** Xin Li, Juan Xu, Jianfeng Wu, Mark H. Weir, Chuanwu Xi

**Affiliations:** † Department of Environmental Health Sciences, School of Public Health, University of Michigan, Ann Arbor, Michigan 48109, United States; ‡ Division of Environmental Health Sciences, College of Public Health, The Ohio State University, Columbus, Ohio 43210, United States; § Sustainability Institute, The Ohio State University, Columbus, Ohio 43210, United States

**Keywords:** COVID-19 lockdown, free-living amoeba, *Legionella* spp., public building, plumbing system, water stagnation

## Abstract

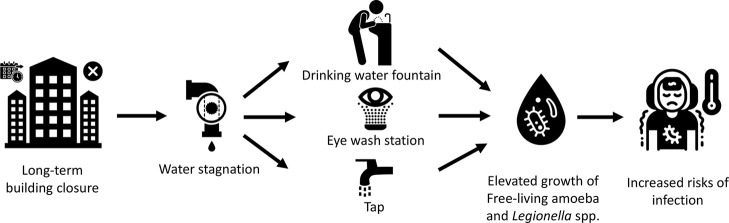

Although water stagnation is widely accepted as an essential factor supporting *Legionella* growth in plumbing systems and “water flashing” has become a common action for water quality control, additional monitoring data in practical spaces are still needed to back up this recommendation. The lockdown of public buildings during the COVID-19 pandemic provided an ideal time window to collect such data on a large scale. This study investigated how the long-term lockdown of a public building and the subsequent water stagnation impact water quality and the population of *Legionella* in water. From June 2020 to May 2021, 192 water samples were collected from a public building during the lockdown and reopening due to the COVID-19 pandemic. Each water sample was assessed for common physicochemical characteristics. Concentrations of *Legionella* and three species of free-living amoeba (FLA) (*Acanthamoeba* spp., *Naegleria fowleri*, and *Hartmannella vermiformis*) were monitored by qPCR. The data suggest that long-term stagnation promotes the population of *Legionella* spp., *Acanthamoeba* spp., and *N. fowleri*. Notable associations were observed between *Legionella* and FLA. These relationships were impacted by stagnation. These results provide important evidence that can inform future water quality management actions to minimize the risk of *Legionella* outbreaks by avoiding the occurrence of water stagnation.

## Highlights

- Long-term stagnation supports the growth of the *Legionella* population in water plumbing systems.
- Avoiding stagnation and regular water flushing help maintain good water quality and decrease the risk of *Legionella* outbreaks.

## 1 Introduction

*Legionella* spp. are opportunistic pathogens ubiquitously present in constructed water systems and equipment such as respiratory therapy equipment, humidifiers, showers, and cooling towers. For immunocompromised and immunosuppressed individuals, inhaling contaminated aerosols can lead to Legionnaires’ disease (LD), Pontiac fever, and even death. Despite improved public awareness, the incidence and outbreak of *Legionella* infections remain high.^1^ In the US, *Legionella* is one of the leading causes of drinking water disease burden due to its outbreaks within healthcare settings and relatively high mortality rate.^2^ About 10,000 LD cases were reported in the United States in 2018, and the number is still increasing.^3^ In Europe and Australia, about 10–15 cases are detected per million individuals in the population per year.^4^ A recent study estimated that the real number of LD cases might be 1.8–2.7 times higher than reported,^5^ which highlights the urgency of this public health threat. Therefore, understanding the environmental and operational factors that affect the survival and growth of *Legionella* spp. in plumbing systems is critical for managing water quality and preventing *Legionella* outbreaks.

It seems common knowledge that stagnation supports the growth of *Legionella*. However, the available scientific evidence is more complicated and less convincing than what is frequently conveyed in peer-reviewed literature, *Legionella* control guidelines, news media, and social media.^6^ Previous publications have provided evidence for the following aspects: (i) avoiding stagnation of hot water (≥45 °C) and chlorinated water reduced the *Legionella* population,^7,8^ (ii) *Legionella* were more likely to colonize in dead legs and low-use taps,^9,10^ (iii) the removal or renewal of dead legs reduced *Legionella* contamination,^11,12^ (iv) an increase in flow rate reduced the *Legionella* population in water,^8,13^ and (v) the seasonal operation of hotels was a principal predicting factor for *Legionella* colonization.^14^ However, these studies did not provide a direct attribution of stagnation impacts on *Legionella* growth because stagnation was usually linked to a low flow rate or intermittent water use instead of complete stagnation. In addition, several studies provided conflicting results. For instance, Sidari et al.^15^ reported that removing dead legs had no impact on *Legionella* control; Liu et al.^16^ conducted a five-week, pilot-scale experiment where the completely stagnant conditions had the lowest numbers of biofilm-associated *Legionella* spp.; and Bédard et al.^17^ observed no increase in *Legionella* gene copy numbers in a contaminated building that was completely closed for up to 10 days. Well-designed field studies are needed to provide more evidence regarding the impact of complete stagnation on *Legionella* growth in water plumbing systems.

Prior research has suggested that decreased water consumption heightens the risk of *Legionella* growth and dissemination as well as that of its natural environmental reservoirs, free-living amoebae (FLA).^18−22^ The COVID-19 lockdowns and subsequent reopenings provided us with a good opportunity to observe the impact of the extended stagnation on the growth of *Legionella*. In this study, water samples were taken monthly from different public building outlets during building lockdown, partial reopening, and full occupancy. The impacts of stagnation on *Legionella* growth and water quality were observed. Three species of FLA (*Acanthamoeba* spp., *N. fowleri*, and *H. vermiformis*) were also monitored. The results of this research may provide scientific evidence to develop guidance and practice for controlling *Legionella* in the plumbing systems in buildings.

## 2 Methods and Materials

### 2.1 Sampling

A total of 192 water samples at a volume of 1500 mL were collected monthly from two eye washers, three sink taps, and three drinking water fountains in the School of Public Health building at the University of Michigan from June 2020 to May 2021. During this period, the building was closed from June 2020 to August 2020, partly reopened from September 2020 to January 2021 (most classes were hybrid), and opened to its full occupancy (most classes were in-person) after February 2021. No flushing or chlorination occurred in the building water system during that time. Those 192 water samples included 96 samples that were collected right after the faucets were turned on and were labeled as “the first-liter sample”; the other 96 samples were collected after keeping the water running for 1 min and were labeled as “the second-liter sample” following protocols in the ISO 11731:2017.^23^ For each sample, water physicochemical parameters were measured and recorded within 30 min after the sampling. The temperature, pH value, and oxidation–reduction potential (ORP) were measured using a HANNA HI98121 pH/ORP/temperature combo tester. The electrical conductivity (EC) and total dissolved solids (TDS) were measured using a HoneForest TDS meter. The total chlorine and free chlorine concentrations were measured using the HACH chlorine test kit and the HACH Pocket Colorimeter II.

### 2.2 Quantification of *Legionella* spp. and FLA

#### 2.2.1 DNA Extraction

To detect *Legionella* spp. and FLA separately, 1000 mL of each water sample was vacuum filtered through a mixed ester cellulose membrane filter (Fisherbrand; pore size 0.45 μm). Each membrane filter was cut into small pieces for DNA extraction and subsequent qPCR analysis. DNA extraction was performed with the RNeasy PowerMicrobiome Kit (QIAGEN, Germany) according to the manufacturer’s instructions. Extracted DNA samples were kept at −20 °C for future analysis.

#### 2.2.2 Quantitative PCR Assays

The primers and probes used in the qPCR assay and the detection limits are summarized in Table S1. *Legionella* genus-specific primers Leg23SF/R and a VIC-labeled probe Lsp23SP targeting the 23S rRNA gene were used to quantify all *Legionella* spp. by the TaqMan method.^24^ Three FLA species were detected by using primers and probes targeting the 18S rRNA gene. To detect all *Acanthamoeba* spp., primers AcnatF900 and AcantR1100 and a Cy5-labeled probe AcantP1000 were used in the TaqMan method.^25^ To detect *N. fowleri*, primers NaeglF192 and NaeglR344 and a HEX-labeled probe NfowlP were used in the TaqMan method.^25^ To detect *H. vermiformis*, primers Hv1227F and Hv1728R were used in the SYBR Green method.^26^

For the SYBR assay, each real-time PCR reaction contained 1x QIAGEN QuantiTect Probe PCR Master Mix, 0.5 μmol/L of each primer, 0.5 μmol/L of each probe, and 3 μL of DNA in a 20 μL reaction volume. PCRs were performed in a Mastercycler RealPlex2 instrument (Eppendorf, Germany) with one initial hold at 95 °C for 15 min, followed by 40 cycles at 94 °C for 15 s, 61 °C for 30 s, and 72 °C for 30 s. Fluorescence was measured at the end of each 72 °C incubation. The results were analyzed using Mastercycler ep realplex software.

For the TaqMan assay, each real-time PCR reaction contained 1x QIAGEN QuantiTect Probe PCR Master Mix, 0.2 μmol/L of each primer, 0.2 μmol/L of each probe, and 3 μL of DNA in a 20 μL reaction volume. PCRs were performed in a Mastercycler RealPlex2 instrument (Eppendorf, Germany) with one initial hold at 95 °C for 15 min, followed by 40 cycles at 95 °C for 15 s and 60 °C for 60 s. Fluorescence was measured at the end of each 60 °C incubation. The results were analyzed using the Mastercycler ep realplex software.

Genomic DNA of the *Legionella pneumophila* subsp. *pneumophila* strain Philadelphia-1 (ATCC 33152), the *N. fowleri* strain Carter (ATCC 30174D), the *Acanthamoeba castellanii* (Douglas) strain Page (ATCC 30010D), and the *Hartmannella vermiformis* strain Page (ATCC 50237) were used to build the standard curve and determine the detection limits. Nuclease-free water was used for serial dilution and a negative control. The presence or absence of *Legionella* and three FLA was defined according to the presence of the fluorescent signal in the qPCR method. For concentrations below the quantification limit and above the detection limit, the values were censored and substituted with estimated values using robust order statistics (ROS).

### 2.3 Data Analysis

Statistical analysis was performed using R statistical software (ver. 4.0.5; R Foundation for Statistical Computing, Vienna, Austria). The analysis of the presence or absence of *Legionella* and the three FLA was performed according to the fluorescence signal of the qPCR method. The analysis of the concentrations of *Legionella* and the three FLA was performed based on ROS estimations based on data of samples whose *Legionella* app. levels were up to the detection limit. For all statistical tests, a *p*-value < 0.05 was considered statistically significant.

#### 2.3.1 Assessing the Impacts of Short-Term Water Stagnation

To investigate the impact of short-term water stagnation, samples were divided based on sampling times into two groups: the “first-liter” and “second-liter” samples. The “first-liter” samples were collected immediately after turning on the faucets and represented the stagnated water in the plumbing system, while the “second-liter” samples were collected after keeping the water running for 1 min, representing fresher water entering the plumbing system. The concentrations of targeted microorganisms and the levels of water physicochemical parameters in the “first-liter” and “second-liter” samples were considered dependent; thus, the Wilcoxon signed-rank test was used to compare these parameters between the two groups. Furthermore, the presence or absence of targeted microorganisms between the two sampling times was determined using McNemar’s test.

#### 2.3.2 Assessing the Impacts of Extended Water Stagnation

Due to the COVID-19 pandemic, the School of Public Health building experienced three distinct phases of operation during the sampling period. As a result, the collected water samples were divided into three distinct groups. The “lockdown” group consisted of 48 water samples collected while the building was closed, the “partial reopening” group included 80 samples collected during the partial reopening of the building, and the “full occupancy” group contained 64 samples collected after the building had opened to full occupancy. To visualize the dynamics of the physicochemical parameters, the FLA, and the *Legionella* spp. in each phase of stagnation, line and box plots were utilized. Additionally, the Kruskal–Wallis test was utilized to assess any differences in the targeted microorganism concentrations and water physicochemical parameters among the three phases. Fisher’s exact test was conducted to compare the presence or absence of the targeted microorganisms among the three phases.

#### 2.3.3 Assessing the Effects of FLA on *Legionella* spp. During Extended Water Stagnation

The analysis included FLA hosts, as they are a significant factor in promoting the growth of *Legionella* spp. Furthermore, it has been reported that the population of FLA is linked to water stagnation.^21,22^ Two perspectives were considered when studying the impacts of FLA on *Legionella*: the presence of *Legionella* and the concentrations of *Legionella*. The presence of each FLA was assessed in relation to the presence of *Legionella* using the Pearson chi-squared test. In addition, to investigate the correlation between *Legionella* and FLA concentrations in the water samples, a Spearman’s correlation analysis was performed using ROS estimations data. Multiple linear regression (MLR) was conducted on log-transformed concentrations of *Legionella* to evaluate this correlation further. Stepwise selection in both directions was adopted to select the best model with the lowest AIC value. Then, tests for the multicollinearity and assumptions of MLR were done to ensure that the model was valid.

## 3 Results

To study the impact of extended stagnation on the presence of *Legionella* in the plumbing system in public buildings, we took advantage of the opportunity to collect water samples in a building on the University of Michigan campus when it was completely shut down during the COVID-19 pandemic and then reopened. Our sample campaign covered different phases of the operation of this building: shutdown, partial reopening, and reopening to its full operation. In addition to quantifying the presence of *Legionella* spp., *Acanthamoeba* spp., *N. fowleri,* and *H. vermiformis* in those samples, we also measured regular water physicochemical parameters, including total chlorine, free chlorine, EC, ORP, TDS, pH, and temperature.

### 3.1 Impacts of Water Stagnation on Physicochemical Characteristics

We first looked at the dynamics of water physicochemical parameters and how stagnation impacted them. During the research period, the average values of all water physicochemical parameters were within the normal range of drinking water quality standards. However, fluctuations were observed for some parameters (Figure S1). The Wilcoxon test (Table 1) revealed distinct variations in the water characteristics between the “first-liter” and “second-liter” samples. During the three stagnation phases, the “first-liter” samples had notably lower total chlorine levels, with *p*-values of 0.02, <0.01, and <0.01, respectively, while no significant differences for free chlorine were observed. In the “partial reopening” and “full occupancy” phases, the “first-liter” samples had lower ORP values, with *p*-values of 0.01 and 0.02, and lower pH values, with *p*-values <0.01. The “first-liter” samples in the “lockdown” phase tended to have significantly lower temperatures than the “second-liter” samples (*p*-value < 0.01), while the opposite was true in the other two phases, with *p*-values of <0.01 and 0.02. No significant differences in the physicochemical water parameters were detected among water samples from various tap types and floors.

**Table 1:** Different Phases of Stagnation in Water Physicochemical Parameters, *Legionella* spp., and FLA^a^

	sample time	lockdown, mean (SD)	partial reopening, mean (SD)	full occupancy, mean (SD)	Kruskal–Wallis test (*p*-value)
total chlorine (mg/L)	first-liter	2.33 (2.04)	2.72 (1.94)	3.55 (1.81)	0.05 *
	second-liter	4.03 (2.30)	5.15 (1.96)	5.56 (1.09)	0.02*
	Wilcoxon test	0.02*	<0.01**	<0.01**	
free chlorine (mg/L)	first-liter	0.12 (0.26)	0.16 (0.25)	0.21 (0.28)	0.02*
	second-liter	0.40 (0.78)	0.21 (0.35)	0.17 (0.14)	0.24
	Wilcoxon test	0.22	0.05	0.84	
EC (uS/cm)	first-liter	482.21 (36.42)	529.48 (78.25)	560.38 (109.32)	<0.01**
	second-liter	488.67 (30.79)	502.10 (84.37)	529.09 (107.23)	0.45
	Wilcoxon test	0.52	0.03*	0.09	
ORP (mV)	first-liter	228.96 (59.95)	203.15 (29.16)	209.00 (28.29)	0.41
	second-liter	246.79 (60.10)	215.88 (24.31)	219.69 (29.53)	0.27
	Wilcoxon test	0.10	0.01*	0.02*	
TDS (ppm)	first-liter	225.92 (18.56)	247.68 (38.67)	269.69(63.97)	<0.01**
	second-liter	230.25 (13.44)	237.63 (35.08)	258.19 (64.05)	0.23
	Wilcoxon test	0.35	0.04*	0.14	
pH	first-liter	9.12 (0.45)	9.36 (0.11)	9.30 (0.13)	0.13
	second-liter	9.14 (0.51)	9.44 (0.11)	9.43 (0.11)	0.25
	Wilcoxon test	0.35	<0.01**	<0.01**	
temperature (°C)	first-liter	21.71 (4.78)	23.24 (2.40)	22.75 (2.97)	0.91
	second-liter	24.49 (2.03)	21.54 (3.39)	20.68 (3.51)	<0.01**
	Wilcoxon test	<0.01**	<0.01**	0.02*	
Gene Copies/100 mL					
*Legionella* spp.	first-liter	340 (824)	136 (273)	104 (176)	<0.01**
	second-liter	419 (869)	65 (148)	90 (116)	0.09
	Wilcoxon test	0.68	0.03*	0.37	
*N. fowleri*	first-liter	23 (29)	8 (17)	1 (1)	<0.01**
	second-liter	30 (35)	6 (9)	1 (1)	<0.01**
	Wilcoxon test	0.10	0.58	0.44	
*Acanthamoeba* spp.	first-liter	1,027 (3,071)	3,416 (10,151)	64 (86)	<0.01**
	second-liter	325 (580)	667 (1,862)	25 (47)	<0.01**
	Wilcoxon test	0.30	<0.01**	0.02*	
*H. vermiformis*	first-liter	4,407 (21,058)	31 (52)	794 (4,289)	0.70
	second-liter	1,520 (6,499)	1,135,500 (7,131,600)	351 (1,677)	0.59
	Wilcoxon test	0.91	0.96	0.70	

a A *p*-value of < 0.01 is denoted by ** and a *p*-value of < 0.05 is denoted by *.

The effects of prolonged stagnation are demonstrated in the box plots of Figure S2 and Table 1, which show that the closure of the building had a notable effect on most water physicochemical parameters, especially in the “first-liter” samples. Particularly, the “lockdown” phase exhibited the lowest total chlorine (*p*-value = 0.05), free chlorine (*p*-value = 0.02), EC (*p*-value < 0.01), and TDS (*p*-value < 0.01) levels in the “first-liter” samples. Additionally, the “second-liter” samples in the same phase revealed the lowest total chlorine (*p*-value = 0.02) and the highest temperatures (*p*-value < 0.01). Meanwhile, the ORP and pH values remained consistent across the different phases for both the “first-liter” and “second-liter” samples.

### 3.2 Prevalence of *Legionella* and FLA and Impacts of Water Stagnation

To investigate the impact of water stagnation on the growth of *Legionella* and FLA, we quantified their gene copies in each water sample (Table S2). *Legionella* were detected in 68% of the water samples, with concentrations ranging from 10 (gene copies/100 mL) to 3,200 (gene copies/100 mL) with an average of 170 (gene copies/100 mL). Among the three species of FLA, *N. fowleri* were the most frequently detected (85%), followed by *Acanthamoeba* spp. (73%). However, the highest concentration of *N. fowleri* was only 130 (gene copies/100 mL), and the mean concentration was only 10 (gene copies/100 mL). *H. vermiformis* were detected in 33% of the water samples but had the highest mean concentration (237,500 gene copies/100 mL) among the three species of FLA. Concentrations of *H. vermiformis* ranged wildly from 10 (gene copies/100 mL) to about 45,000,000 (gene copies/100 mL).

The Wilcoxon test was performed to compare the “first-liter” and “second-liter” samples on the populations of *Legionella* and FLA (Table 1). The data show that different sampling times only had significant impacts on the concentrations of *Legionella* spp. in the “partial reopening” phase (*p*-value = 0.03) and on *Acanthamoeba* spp. in both the “partial reopening” (*p*-value < 0.01) and “full occupancy” (*p*-value = 0.02) phases. No significant differences were observed when comparing groups of *N. fowleri* and *H. vermiformis* between different sampling times. Fisher’s exact test showed no significant difference in the presence of tested microorganisms between the “first-liter” and “second-liter” samples (Table 2). No substantial differences in microbial concentrations among water samples from various tap types and floors were observed.

**Table 2:** Positive Rates of *Legionella* spp. and FLA in Water Samples^a^

	sample time	lockdown, positive rate (%)	partial reopening, positive rate (%)	full occupancy, positive pate (%)	Fisher’s exact test (*p*-value)
*Legionella* spp.	first-liter	21	75	97	<0.01**
	second-liter	33	70	88	<0.01**
	Fisher’s exact test	0.52	0.80	0.35	
*N. fowleri*	first-liter	100	73	84	<0.01**
	second-liter	100	70	97	<0.01**
	Fisher’s exact test	-	0.99	0.20	
*Acanthamoeba* spp.	first-liter	79	88	66	0.08
	second-liter	75	90	34	<0.01**
	Fisher’s exact test	0.99	0.99	0.02*	
*H. vermiformis*	first-liter	33	28	38	0.65
	second-liter	42	30	34	0.63
	Fisher’s exact test	0.77	0.99	0.99	

a A *p*-value of < 0.01 is denoted by ** and a *p*-value of < 0.05 is denoted by *. Note: the positive rate (%) is the percentage of samples containing the targeted microorganisms (having fluorescence signals in the qPCR method).

**Figure 1:**
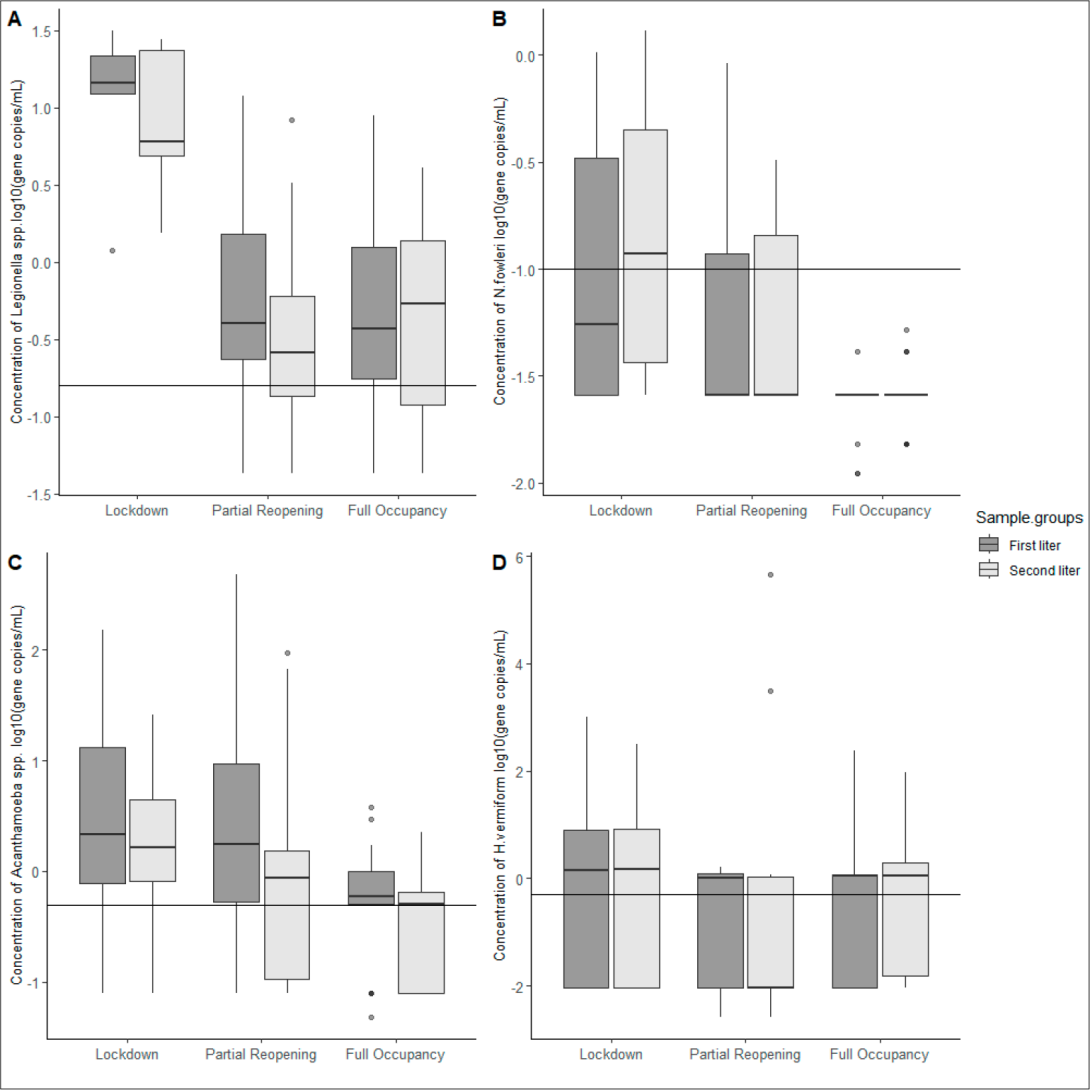
Log-transformed concentrations of *Legionella* spp. and FLA in different sample groups across three water stagnation phases. The long-term stagnation had significant impacts on the population of (A) *Legionella* spp. (*p*-value < 0.01), (B) *N. fowleri* (*p*-value < 0.01), and (C) *Acanthamoeba* spp. (*p*-value < 0.01), but did not impact (D) *H. vermiformis*. The horizontal lines in the plot represent the detection limit of the qPCR method for each microorganism.

In terms of the impact of extended stagnation, data in the box plots (Figure 1) and from the Kruskal–Wallis test (Table 1) showed that different stagnation phases significantly impact the mean concentrations of *Legionella* spp., *N. fowleri*, and *Acanthamoeba* spp. For *Legionella* spp., their concentrations in the “first-liter” samples were significantly higher (*p*-value < 0.01) in the “lockdown” phase than in the other phases; for *N. fowleri*, both the “first-liter” (*p*-value < 0.01) and “second-liter” (*p*-value < 0.01) groups had the highest concentrations in the “lockdown” phase. *Acanthamoeba* spp. tended to have its highest concentration in the “partial reopening” phases for both the “first-liter” (*p*-value < 0.01) and “second-liter” (*p*-value < 0.01) groups, while its concentration in the “lockdown” phase was still significantly higher than that in the “full occupancy” phase. The results of Fisher’s exact test (Table 2) indicate that although the concentrations of *Legionella* spp. in the “lockdown” phase were higher than those in the other phases, the positive rate was the lowest (*p*-value < 0.01).

### 3.3 Relationship between FLA and *Legionella* spp. and the Impacts of Stagnation

The impacts of FLA on *Legionella* were studied from two perspectives: the presence and the concentrations of *Legionella*. Pearson chi-squared test results show that *N. fowleri* (χ^2^ = 8.75, df = 1, *p*-value < 0.01) and *Acanthamoeba* spp. (χ^2^ = 4.77, df = 1, *p*-value = 0.03) are of high significance to the occurrence of *Legionella* (Table S3). A Spearman’s correlation test was conducted based on ROS estimations. The results demonstrate the relationship between the concentrations of targeted microorganisms (Table S4). The analysis found a significant positive correlation between the concentrations of *Legionella* spp. and both *N. fowleri* (r = 0.43, *p*-value < 0.01) and *Acanthamoeba* spp. (r = 0.33, *p*-value < 0.01). The concentrations of *N. fowleri* and *Acanthamoeba* spp. are also positively related (r = 0.22, *p*-value < 0.05).

According to the results of stepwise selection, an MLR model was built with the log-transformed concentrations of *Legionella* spp. as the dependent variable and the concentrations of *N. fowleri* and *Acanthamoeba* spp. as the independent variables. Stagnation phases and the interaction terms between stagnation and FLA were also included in the model (Table 3). After the adjustment for stagnation, the relationships between *Legionella* and FLA became insignificant in the MLR model. Compared with the “lockdown” phase, the “partial reopening” (β = −1.4019, *p*-value < 0.001) and “full occupancy” (β = −1.1177, *p*-value < 0.001) phases tended to have lower *Legionella* spp. The significant *p*-value for the interaction terms suggested that the relationship between *Legionella* spp. and FLA is different for different phases. The supportive impacts of *N. fowleri* (β = 2.3116, *p*-value = 0.0236) and *Acanthamoeba* spp. (β = 0.0164, *p*-value = 0.0160) on *Legionella* growth were greater in the “partial reopening” phase than in the “lockdown” phase.

**Table 3:** Linear Regression Model for the Log-Transformed Concentrations of *Legionella* spp. Based on FLA Concentrations and Stagnation^a^

	coefficient (β)	SE	*t*-value	*p***-**value (>|*t*|)
(intercept)	1.0548	0.1707	6.177	<0.01**
*N.fowleri*	–0.9532	0.8800	–1.083	0.28
*Acanthamoeba* spp.	0.0012	0.0033	0.362	0.72
Stagnation (Reference: “Lockdown”)				
partial reopening	–1.4019	0.1876	–7.473	<0.01**
full occupancy	–1.1177	0.2580	–4.332	<0.01**
N. fowleri * partial reopening	2.3116	1.0056	2.299	0.02*
N. fowleri * full occupancy	–1.1305	7.3024	–0.155	0.88
*Acanthamoeba* spp. * partial reopening	0.0164	0.0067	2.451	0.02*
*Acanthamoeba* spp. * full capacity	–0.0908	0.0950	–0.955	0.34

a A *p*-value of < 0.01 is denoted by ** and a *p*-value of < 0.05 is denoted by *.

## 4 Discussion

Although the culture method is a “gold standard” for the detection of *Legionella* spp. in water, it has many limitations. Culture is a relatively unpleasant environment for microorganisms. The occurrence of amoeba has a significant impact on culture-based methods. The culture-based methods cannot detect the viable but nonculturable (VBNC) form of *Legionella*,^27^ but amoebae can transform VBNC cells into infectious cells.^28^ Besides, amoebae expel bacteria within vesicles. Although amoeba trophozoites can be completely destroyed by freeze–thawing treatment and sonication, bacteria in those vesicles cannot be dispersed.^29^ Cells within one vesicle only form a single colony on the culture plates.^30^ Therefore, the current monitoring method is likely to underestimate the concentration of infectious *Legionella* and the risk of infection. PCR-based methods, such as qPCR, have high sensitivity, specificity, and throughput.^31^ Although DNA from free and dead cells in water could lead to overestimating concentrations,^32^ qPCR can still provide information on the risk level of the occurrence and quantity of *Legionella* and can be used for initial screening and early warning of outbreaks.

This study investigated the influences of water stagnation on water quality and the growth of pathogens of public health concern. It is important to note that the quantification of *Legionella* and FLA relied on the qPCR method, meaning that the concentrations of these microorganisms reflect the number of gene copies detected in water samples rather than the actual number of cells. Therefore, these concentrations serve as indicators of the potential risks associated with these pathogens.

### 4.1 Impacts of Short and Long-Term Water Stagnation

The comparison of two sampling times reveals the effects of short-term water stagnation. Specifically, the “first-liter” samples were collected from a water pipe that had been inactive for a significant period (at least 12 h), while the “second-liter” samples were obtained immediately after letting the water run for 1 min, thus representing fresher water entering the plumbing system. The data indicate that short-term water stagnation may result in decreased levels of total chlorine, ORP, and pH, but an increase in temperature. However, these changes may not significantly impact the population of microorganisms, particularly *Legionella* spp. Moreover, the higher population of *Acanthamoeba* spp. in “second-liter” samples during the “partial reopening” and “full occupancy” phases may be attributed to the shedding of the biofilm caused by increased water flushing.

### 4.2 Impacts of Different Stagnation Phases

The comparison between different stagnation phases demonstrated the significant impact of longer-term stagnation on water physicochemical qualities and microbial growth. The extended stagnation significantly altered the water’s physicochemical characteristics, including total chlorine, free chlorine, temperature, EC, and TDS. The decrease in disinfectant residual concentrations following building closure observed in this study is consistent with previous findings.^33^ The expected lower ORP and higher pH values during the “lockdown” phase were observed as well, although the changes were not significant.

The extended stagnation also supported the growth of microorganisms, including *Legionella* spp., *N. fowleri*, and *Acanthamoeba* spp. in the plumbing system (Figure 1). The increase of *Acanthamoeba* spp. concentrations during the “partial reopening” phase and the return to low concentrations over time demonstrate that stagnation alone is not a continuing process but rather a dynamic one (Table 1). This may be relative to the entrance and exit from biofilms over the course of stagnancy in addition to the small-scale sloughing that was possible when the samples were taken. In addition, the discrepancy between the concentrations and the positive rates of *Legionella* spp. suggests that a decrease in water flushing during the “lockdown” phase may have led to a lower likelihood of bacteria detaching from the biofilm but a higher concentration of bacteria per detachment event.

The results of the Pearson chi-squared test demonstrating the co-occurrence of *Legionella* spp., *Acanthamoeba* spp., and *N. fowleri* are consistent with previous studies,^34,35^ which indicates that the FLA could protect or promote the growth of *Legionella* spp. in water distribution systems. Moreover, the Spearman’s correlation test results also support the fact that *N. fowleri* and *Acanthamoeba* spp. are two significant hosts for *Legionella* spp. However, after adjustment for stagnation, the relationships became not robust, which indicates that the impact of stagnation may play a more essential role than the hosting of FLA in the population of *Legionella* (Table 3). Furthermore, the data suggest that the sudden increase in water usage during the “partial reopening” phase may have a more significant impact on the interaction between *Legionella* spp. and FLA compared to the “full occupancy” phase.

### 4.3 Limitations of This Study

There are some limitations of this study that we need to admit. *Legionella* was measured at the genus level, even though *L. pneumophila* is the primary pathogenic bacterium affecting humans. Additionally, qPCR technology cannot distinguish between living organisms and dead organisms. As a result, the potential impacts of water stagnation on living microorganisms and the infection risks have gone undetected. Future research could employ advanced techniques like PMA-qPCR and DVC-FISH to explore the relationships between living *Legionella* and living FLA. Furthermore, monitoring a broader range of microorganisms and water quality parameters could help develop a more comprehensive model of *Legionella* growth in water systems.

## 5 Conclusions

The results of this study support the hypothesis that long-term stagnation can significantly degrade water quality and increase the population of microorganisms in water plumbing systems. Although we were unable to determine the exact duration of stagnation required for a noticeable increase of the *Legionella* spp. and FLA populations in plumbing systems because the first group of samples was collected three months after the building shutdown, our current data provide sufficient evidence that increasing water flashing can benefit the management of water quality and microorganism levels. Therefore, the following measures can be adopted to mitigate the risk of *Legionella* spp. in plumbing systems: (1) avoiding long-term water stagnation; (2) being aware of fluctuations in water qualities and bacteria populations after reopening, as it may take longer than expected for water physicochemical characteristics and bacteria populations to return to normal; and (3) controlling *N. fowleri* and *Acanthamoeba* spp. growth to help manage the population of *Legionella* spp.

## Supporting Information Available

Figure S1, water physicochemical parameters in water samples over time; Figure S2, water physicochemical parameters in different sample groups across three water stagnation phases; Table S1, primers and probes for the qPCR assay; Table S2, descriptive statistics of *Legionella* spp. and FLA; Table S3, Pearson’s chi-squared test for *Legionella* spp. and FLA; and Table S4, Spearman’s correlation matrix for FLA and *Legionella* spp. concentrations (PDF)
