# Supplementary material for: Prevalence of Legionella in a Public Building Water Plumbing System During COVID-19 Lockdown
Source: Environ Health (Wash). 2023 Oct 4;1(5):352–9. doi: 10.1021/envhealth.3c00058 (PMC11503721; doi:10.1021/envhealth.3c00058)
Supplement: Supplementary file 1 — eh3c00058_si_001.pdf [file eh3c00058_si_001.pdf]

Supporting Information

Prevalence of *Legionella* in a public building water plumbing system during COVID-19 lockdown

Xin Li [a], Juan Xu [b], Jianfeng Wu [a], Mark H. Weir [b,c], and Chuanwu Xi [a*]

[a] *Department of Environmental Health Sciences, School of Public health, University of Michigan, Ann Arbor, Michigan 48109, United States*

[b] *Division of Environmental Health Sciences, College of Public Health, The Ohio State University, Columbus, Ohio 43210, United States*

[c] *Sustainability Institute, The Ohio State University, Columbus, Ohio 43210, United States*

*E-mail: cxi@umich.edu

The water physicochemical parameters during the research period

Line plots were created to describe the water physicochemical parameters over time during the research period (Figure 1S). Each line represents a sample site. The concentration of total chlorine increased slightly from June 2020 to May 2021. The concentration of FCL, the EC levels, the TDS levels, and the ORP levels have similar trends. They first decreased and then increased after October 2021. On the contrary, the pH values first increased before October 2021 and then decreased. The temperature of first-liter samples was stable over time, while the temperature of second-liter samples increased before July 2020 and then decreased.

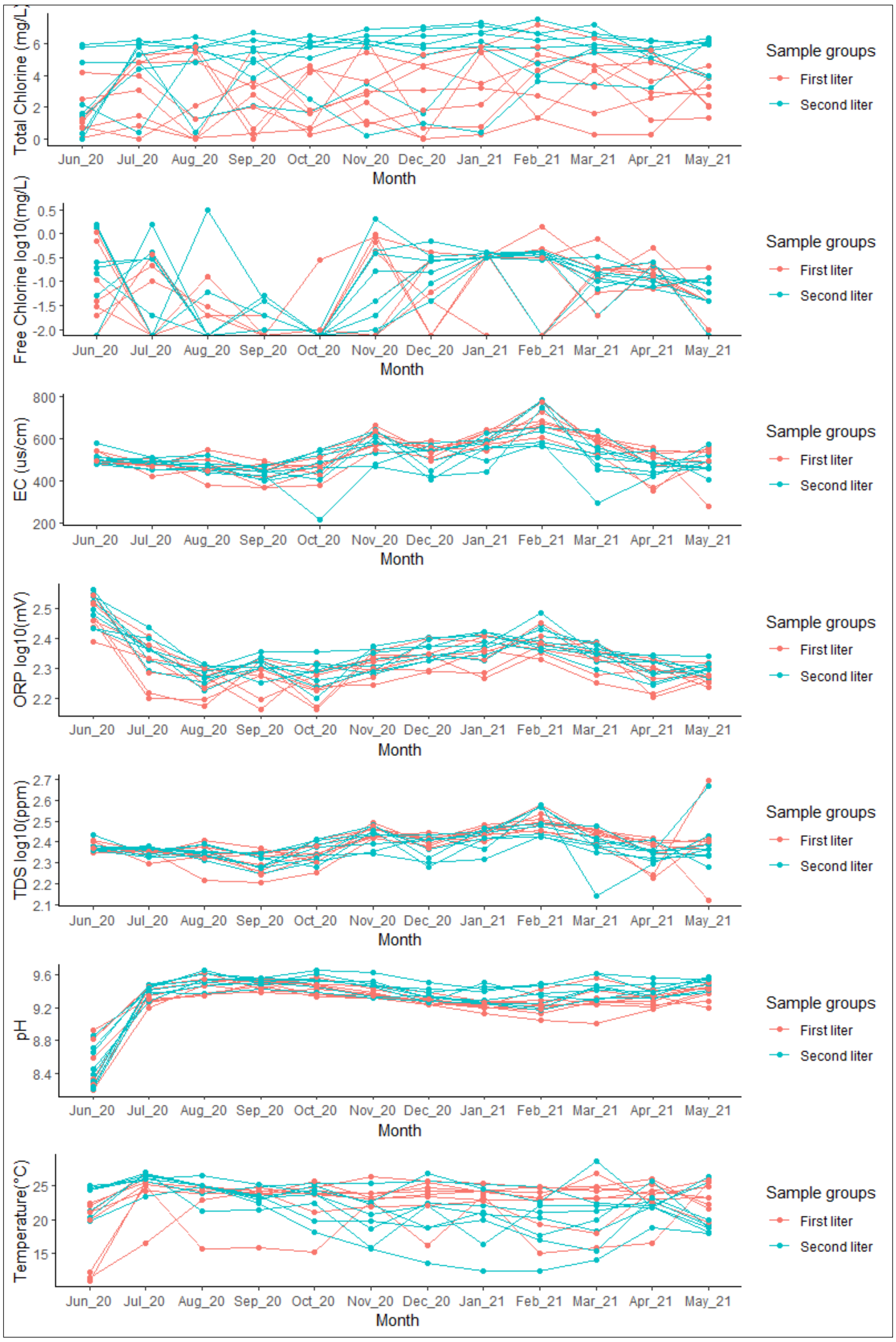

Figure S1. The water physicochemical parameters in water samples over time.

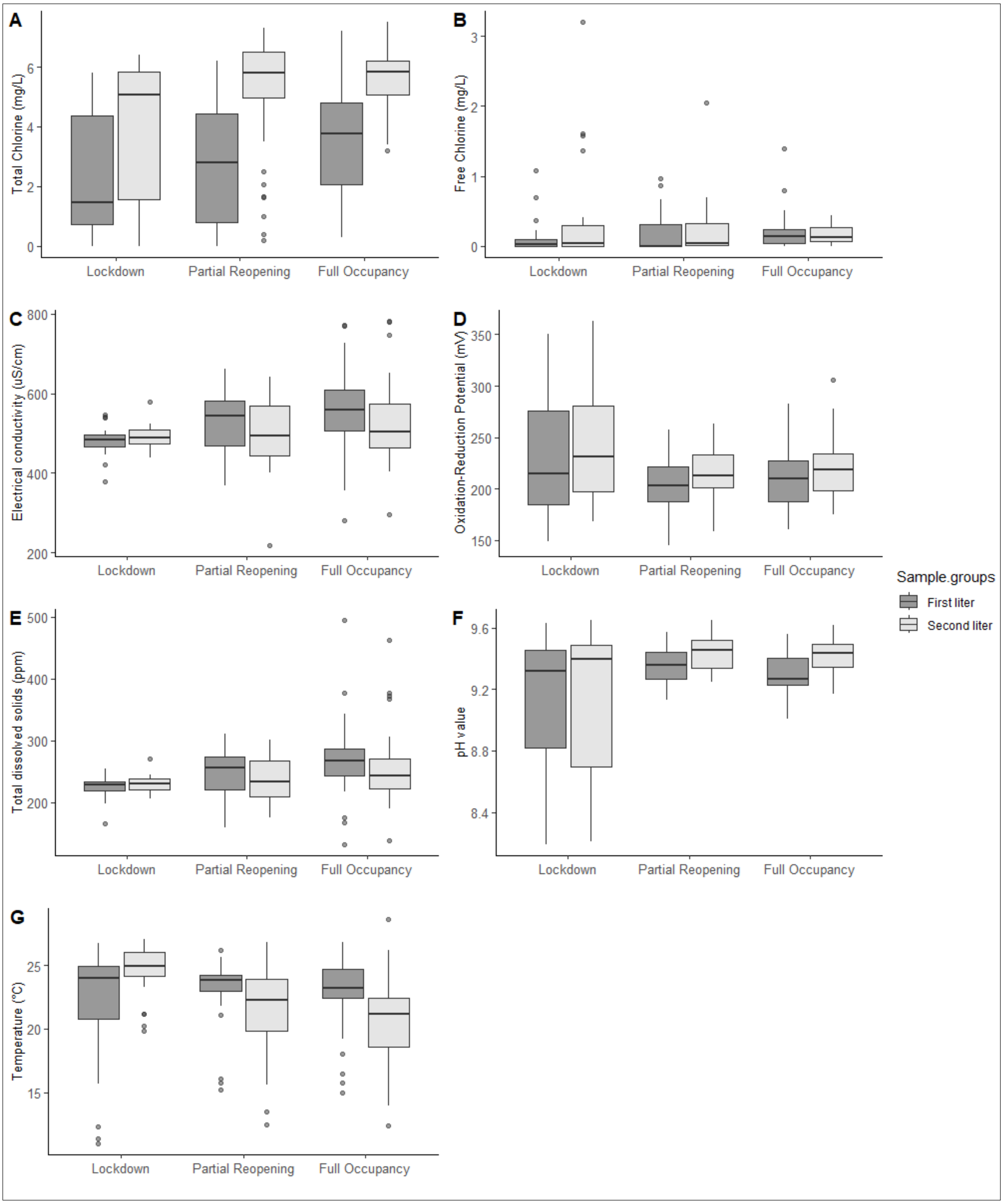

Figure S2. Water physicochemical parameters in different sample groups across three water stagnation phases. Significant differences between "first-liter" and "second-liter" samples were observed for (A) total chlorine, (D) ORP, (F) pH and (G) temperature. Long-term stagnation had significant impacts on (A) total chlorine, (B) free chlorine, (C) EC, (E) TDS and (G) temperature.

Table S1. Primers and probes for the qPCR assay.

Species	Primer or probe	Nucleotide sequence (5′-3′)	Gene targeted	Detection limit (gene copies/µL)	Ref.
***Legionella* spp.**	Leg23SF	CCCATGAAGCCCGTTGAA	23S rRNA (92 bp)	1.6 (corresponded with 0.16 gene copies/mL water samples)	1
	Leg23SR	ACAATCAGCCAATTAGTACGAGTTAGC			
	Lsp23SP VIC-TAMRA	TCCACACCTCGCCTATCAACGTCGTAGT			
***Acanthamoeba* spp.**	Lsp23SP VIC-TAMRA	TCCACACCTCGCCTATCAACGTCGTAGT	18S rRNA (180 bp)	1 (corresponded with 0.16 gene copies/mL water samples)	2
	AcantR1100	TAAATATTAATGCCCCCAACTATCC			
	Cy5-labeled AcantP1000	Cy5-CTGCCACCGAATACATTAGCATGG-BHQ3			
N. fowleri	NaeglF192	GTGCTGAAACCTAGCTATTGTAACTCAGT	18S rRNA (153 bp)	5 (corresponded with 0.16 gene copies/mL water samples)	2
	NaeglR344	CACTAGAAAAAGCAAACCTGAAAGG			
	HEX-labeled NfowlP	HEX-ATAGCAATATATTCAGGGGAGCTGGGC-BHQ1			
H.vermiformis	Hv1227F	TTACGAGGTCAGGACACTGT	18S rRNA (502 bp)	10 (corresponded with 0.16 gene copies/mL water samples)	3
	Hv1728R	GACCATCCGGAGTTCTCG			

Table S2. Descriptive statistics of *Legionella* spp. and FLA

Unit: gene copies/mL		*Legionella* spp.	*Acanthamoeba* spp.	*N. fowleri*	*H. vermiformis*
**Overall samples**	Mean	1.68	10.29	0.10	2,375.02
	Median	0.20	0.56	0.03	0.00
	SD	4.64	49.54	0.20	32,555.26
	Positive rate	68%	73%	85%	33%

Table S3. Pearson's Chi-squared test for *Legionella* spp. and FLA.

		Legionella spp. Present	*Legionella* spp. Absent	df	χ^2	p-value
Acanthamoeba spp.	Present	88	52	1	4.7749	0.0289*
	Absent	42	10			
N. fowleri	Present	103	60	1	8.7541	0.0031**
	Absent	27	2			
H. vermiformis	Present	44	20	1	0.0030	0.9565
	Absent	86	42			

Note: the presence or absence of Legionella and three FLA was defined according to the presence of the fluorescent signal in the qPCR method. p<0.01**; p<0.05*

Table S4. Spearman's correlation matrix for FLA and *Legionella* spp. concentrations.

	H. vermiformis	***N. fowleri***	***Acanthamoeba* spp.**
N. fowleri	0.2278		
Acanthamoeba spp.	0.1105	0.2240*	
Legionella spp.	-0.0498	0.4277**	0.3291**

$p<0.01$**; $p<0.05$*

Reference

(1) Nazarian, E. J.; Bopp, D. J.; Saylors, A.; Limberger, R. J.; Musser, K. A. Design and Implementation of a Protocol for the Detection of Legionella in Clinical and Environmental Samples. *Diagnostic Microbiology and Infectious Disease* **2008**, *62* (2), 125–132. https://doi.org/10.1016/j.diagmicrobio.2008.05.004.

(2) Qvarnstrom, Y.; Visvesvara, G. S.; Sriram, R.; Silva, A. J. da. Multiplex Real-Time PCR Assay for Simultaneous Detection of Acanthamoeba Spp., Balamuthia Mandrillaris, and Naegleria Fowleri. *Journal of Clinical Microbiology* **2006**, *44* (10), 3589–3595. https://doi.org/10.1128/JCM.00875-06.

(3) Kuiper, M. W.; Valster, R. M.; Wullings, B. A.; Boonstra, H.; Smidt, H.; van der Kooij, D. Quantitative Detection of the Free-Living Amoeba Hartmannella Vermiformis in Surface Water by Using Real-Time PCR. *Appl. Environ. Microbiol.* **2006**, *72* (9), 5750–5756. https://doi.org/10.1128/AEM.00085-06.
